# Hepatitis B Virus e Antigen in Mother-to-Child Transmission and Clinical Management of Hepatitis B

**DOI:** 10.3390/v17111484

**Published:** 2025-11-08

**Authors:** Qiqi Ning, Jing-hsiung James Ou

**Affiliations:** Department of Molecular Microbiology and Immunology, Keck School of Medicine, University of Southern California, Los Angeles, CA 90033, USA; qiqining@usc.edu

**Keywords:** HBV e antigen, mother-to-child transmission, immune tolerance, persistent HBV replication

## Abstract

Chronic hepatitis B virus (HBV) infection is a major health problem that leads to approximately one million deaths every year worldwide. Mother-to-child transmission (MTCT) is the major cause of chronic HBV infection. HBV e antigen (HBeAg) is a secretory viral protein and modulates the immunological landscape of the newborn to promote HBV persistence. HBeAg actively reprograms innate and adaptive immunity. Mechanistically, HBeAg regulates macrophage polarization, suppresses dendritic cell and natural killer (NK) cell activities, impairs T cell and B cell functions, and promotes the expansion of myeloid-derived suppressor cells (MDSCs). These multifaceted effects contribute to immune tolerance and persistent HBV infection in the offspring of carrier mothers. Clinically, HBeAg status is a critical determinant for MTCT risk stratification and intervention, particularly in resource-limited settings. Despite advances in neonatal immunoprophylaxis and maternal antiviral therapy, residual transmission of HBV persists. Emerging approaches targeting HBeAg directly or restoring antiviral immunity offer promising avenues for breaking immune tolerance and achieving HBV elimination. This review summarizes current understanding of HBeAg-mediated immune modulation and highlights strategies that are being used to disrupt MTCT and treat HBV patients.

## 1. Introduction

Hepatitis B virus (HBV) is a major global health problem. As of 2022, an estimated 257 million individuals worldwide are chronically infected with HBV [1]. In highly endemic regions such as the Western Pacific and sub-Saharan Africa, mother-to-child transmission (MTCT) represents the predominant route of HBV acquisition and chronic HBV infection. HBV e antigen (HBeAg), a well-established marker of active viral replication and immune tolerance, is associated with a high risk of chronic HBV infection after its MTCT [2]. Infants born to HBeAg-positive carrier mothers have a 70–90% risk of developing chronic HBV infection if there is no immunoprophylactic intervention (i.e., administration of HBV vaccine and hepatitis B immunoglobulin [HBIG] at birth) [3,4]. This risk is significantly higher than those born to HBeAg-negative mothers. This exceptionally high rate of chronicity after MTCT underscores the importance of interrupting this pathway for the global elimination of HBV. Neonatal immunoprophylaxis, as mentioned above, has been very successful in disrupting MTCT and reducing the HBV carrier populations, as seen in the studies conducted in Taiwan, where the HBV surface antigen (HBsAg) positivity rate among pregnant women was found to be significantly reduced 32 years after the implementation of the universal infant immunoprophylaxis program in 1984 [5]. High maternal HBeAg level during pregnancy is often associated with high viral load, which may facilitate the MTCT of HBV and its subsequent establishment of chronic infection in neonates. However, accumulating evidence indicates that HBeAg also has immunomodulatory functions that can promote viral persistence. This review will focus on how HBeAg interacts with immune cells and how these interactions promote chronic HBV infection after its MTCT.

## 2. Biological Characteristics and Immunoregulatory Functions of HBeAg

### 2.1. Structure and Function of HBeAg

The HBV genome is a small 3.2-kb DNA molecule that contains four genes: S, P, X, and C. The S gene codes for the viral envelope proteins commonly referred to as surface antigens (HBsAg). P and X genes code for the viral DNA polymerase and the X protein, respectively. The X protein is a regulatory protein that has multiple functions. The C gene codes for two co-carboxy-terminal proteins: the core protein and the precore protein. The core protein is a structural protein that forms the viral core particle. The precore protein contains the entire core protein sequence along with an N-terminal extension of 29 amino acids, termed the precore sequence. It is the precursor of HBeAg found in the serum of HBV patients. The first 19 residues of the precore protein act as a signal peptide, directing the precore protein into the endoplasmic reticulum (ER) [6], where it is cleaved by the signal peptidase to produce a 22 kDa intermediate known as p22. This intermediate can either be relocated back into the cytosol or be transported through the ER and the Golgi apparatus, where it undergoes further processing by a furin-like protease at multiple sites in its C-terminal arginine-rich domain [7]. The final secreted product forms a homodimer [8]; displays the e antigenic determinant (i.e., HBeAg) [6]; and, unlike the core protein, does not bind nucleic acid. The precore protein derivative p22 may inhibit the encapsidation of the HBV pregenomic RNA (pgRNA) [9,10,11]. The secreted HBeAg has immunoregulatory functions [12,13,14]. HBeAg is typically detectable in the serum of patients during the early phase of infection and can serve as a surrogate marker for active viral replication [15,16,17].

### 2.2. Mechanisms of HBeAg-Induced Immune Tolerance

#### 2.2.1. Routes and Impact of HBeAg Exposure on Offspring

Although several studies reported the presence of HBV DNA in oocytes, spermatozoa, and early embryos from HBV-infected individuals [18,19,20,21,22], there is currently no evidence that HBV DNA in these germ cells or early embryos plays any role in MTCT of HBV. In contrast, multiple studies indicated the presence of HBeAg in a significant proportion of neonates born to HBeAg-positive mothers, suggesting possible in utero exposure to HBeAg [17,23,24,25,26]. This prenatal exposure to HBeAg was thought to be important for immune tolerance to HBV and for the establishment of chronic HBV infection after birth [17].

The placenta serves as a filter for the exchange of nutrients and waste between the mother and the fetus. It also provides a physical barrier to protect the fetus from harmful substances in the mother’s blood. How maternal HBeAg may penetrate the placenta to reach the fetus is unclear. The possibility that maternal HBeAg bound to IgG may undergo transcytosis via the neonatal Fc receptor (FcRn) to traverse the placental barrier had been proposed [27]. Alternatively, leakage resulting from compromised placental integrity may also allow HBeAg to reach the fetus [28,29]. The dynamic architecture of the placenta and variability in maternal–fetal circulation likely contribute to heterogeneity in fetal antigen exposure, potentially explaining the variability in MTCT outcomes, even among HBeAg-positive pregnancies.

Labor-induced microtransfusions and mucosal exposure through breastfeeding may also result in exposure to maternal HBV and associated HBV antigens [30,31,32]. Although these exposure routes do not significantly increase MTCT risk under adequate prophylaxis, their cumulative immunological influence on T cell priming, B cell maturation, and antigen-specific tolerance has not been fully elucidated. Longitudinal tracking of vaccine responses in such cohorts may offer new insights into postnatal immune plasticity.

#### 2.2.2. Integrated Immune Programming and Reprogramming

The cumulative effects of in utero, perinatal, and early postnatal exposure to HBeAg likely form a continuous antigenic landscape that shapes the developing immune system of the offspring [17,23,24,25,26,30,31,32]. Rather than acting in isolation, these exposures may synergize to reinforce central and peripheral tolerance mechanisms, leading to durable impairment in HBV-specific immune responsiveness. Notably, neonates exposed to HBeAg across multiple developmental windows have been shown to exhibit blunted T cell responses and altered cytokine production and B cell maturation patterns. These findings suggest that the timing, duration, and intensity of HBeAg exposure are critical determinants of the immunological outcome. This layered exposure model supports the hypothesis that early life antigen contact under non-inflammatory conditions leads to tolerance rather than priming, laying the foundation for the immune reprogramming [17,33].

**Figure 1:**
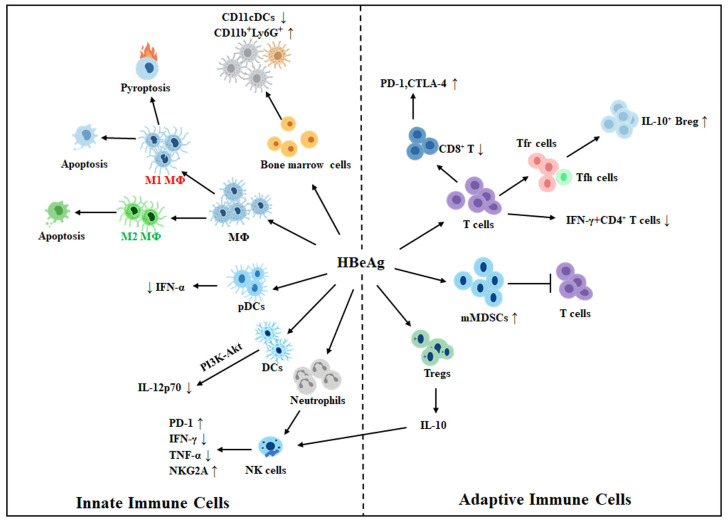
**Effects of HBeAg on innate and adaptive immune cells.** HBeAg may induce macrophages (MΦ) to undergo M1-like proinflammatory polarization and trigger their pyroptosis and apoptosis, or induce them to undergo M2-like anti-inflammatory polarization and induce only apoptosis. HBeAg also suppresses the expression of IL-12p70 in dendritic cells (DCs), likely via the PI3K-AKT pathway. It also suppresses the CpG-induced expression of IFN-α by plasmacytoid DCs (pDCs), increases CD11b^+^Ly6G^+^ immature myeloid cells, and reduces CD11c^+^ CDs. HBeAg also activates neutrophils to suppress natural killer (NK) cells via the induction of PD-1 expression and suppression of IFN-γ and TNF-α in NK cells. It also stimulates regulatory T cells (Tregs) to express IL-10, which induces the expression of inhibitory receptor NKG2A in NK cells. HBeAg positivity is also associated with an increased expression level of PD-1 and CTLA-4 in HBV-specific CD8^+^ T cells and a reduction in CD8^+^ T cells and HBV-specific IFN-γ^+^ CD4+ T cells. Its positivity also increases the ratio of Tfr cells to Tfh cells and is associated with an increased number of IL-10^+^ regulatory B cells (Bregs). HBeAg also induces the expansion of monocytic myeloid-derived suppressor cells (mMDSCs) to suppress T cell activities. Note that innate and adaptive immune cells are presented separately for clarity. However, there is extensive crosstalk, such as through cytokine signaling and antigen presentation, between these two types of cells. ↑, upregulation; ↓, downregulation.

HBeAg orchestrates a complex reshaping of both innate and adaptive immunity, contributing to viral immune evasion and long-term persistence of HBV. It alters host anti-HBV immune responses via multiple mechanisms that involve the alteration of signaling responses, metabolism, epigenetics, and cell-to-cell interactions. It had also been shown to suppress the T cell responses to the core protein, likely by impairing antigen-presenting cells, including dendritic cells, to affect T cell priming [34,35]. The direct effects of HBeAg on innate and adaptive immune cells are illustrated in Figure 1 and will be described in detail in the sections below.

#### 2.2.3. Modulation of Innate Immune Cells

##### Macrophages

Macrophages exhibit phenotypic plasticity, enabling them to dynamically reprogram their activities in response to external stimuli. Depending on microenvironmental cues, macrophages may undergo the M1 pro-inflammatory polarization to produce pro-inflammatory cytokines such as TNF-α and IL-1β, or undergo the M2 anti-inflammatory polarization, characterized by the expression of cellular factors like arginase-1, which depletes arginine in cells and suppresses the production of nitric oxide, and the anti-inflammatory cytokine IL-10 [36,37]. HBeAg can regulate the biological activities of macrophages, thereby contributing to the establishment of an immunosuppressive hepatic microenvironment that facilitates HBV persistence. HBeAg can induce M1-like or M2-like polarization of macrophages.

Pro-inflammatory Activation: HBeAg can stimulate naïve macrophages, including hepatic macrophages (i.e., Kupffer cells), to produce proinflammatory cytokines, including TNF-α, IL-1β, and IL-6 via binding to and activating Toll-like receptor 4 (TLR4) [38,39]. M1 macrophages often display high glycolytic and low oxidative phosphorylation (OXPHOS) activities [40,41,42]. Interestingly, macrophages induced by HBeAg to undergo the M1-like polarization display the opposite metabolic profile with high OXPHOS and low glycolytic activities [38,43]. This atypical metabolism is induced by HBeAg via the TLR4-death receptor 5 (DR5)-death-associated protein 3 (DAP3) axis [38]. The increase in OXPHOS is important for reducing the expression level of IL-1β, as the suppression of OXPHOS enhances the production of IL-1β [38,44]. IL-1β can suppress the transcription of HBV RNAs in hepatocytes by downregulating the transcription factors PPARα and FOXO3, which are critical for HBV RNA transcription [43], and upregulating the expression of the activation-induced cytidine deaminase (AID), which causes HBV DNA mutations [44]. Hence, this rewiring of macrophage metabolism by HBeAg is apparently important for HBV to attenuate the antiviral activities of M1 macrophages. HBeAg has also been shown to upregulate the expression of miR-155 via phosphatidylinositol-3 kinase (PI3K) and NF-κB [45], and induce the expression of Regulator of G protein signaling 16 (RGS16) via the TLR2–p38–STAT5 signaling axis [46]. miR-155 suppresses the expression of BCL-6, SHIP-1, and SOCS1, key negative regulators of the immune signaling pathways. The phosphorylation of RGS16 at Tyr-168 enhances the activation of the ERK pathway as well as macrophage proliferation and migration. Both miR-155 and RGS16 enhance the production of TNF-α and IL-6, which may contribute to HBV pathogenesis [45]. In patients with chronic HBV infection, it was found that HBeAg positivity was associated with M1-type CD14^+^ macrophages and Th-17 cells [47]. It was suggested that these M1 macrophages might promote Th17 cell differentiation and IL-17A secretion, thereby exacerbating hepatic inflammation and fibrosis [47].

Anti-inflammatory activation: Besides the induction of M1-like polarization, HBeAg can also induce macrophages, particularly those that have been exposed to maternal HBeAg, to undergo the M2-like anti-inflammatory polarization characterized by reduced expression of TNF-α and HLA-DR and increased expression of IL-10 and PD-L1, thereby contributing to the establishment of an immunosuppressive microenvironment [38,39,43,48]. HBeAg was shown to upregulate the deacetylase SIRT1, leading to the deacetylation of Notch1, activation of the Akt pathway, and suppression of NF-κB signaling. These molecular events drove the polarization of macrophages toward an M2-like immunosuppressive phenotype and blunted the HBV-specific CD8^+^ T cell response [39,48]. These findings were consistent with the observation that M2 macrophages were detected in the liver of chronic HBV patients [49]. The injection of C57BL/6 mice with the DNA plasmid pAAV/1.2HBV DNA, which contains the 1.2mer HBV genome in an adeno-associated virus (AAV) vector, can lead to persistent HBV replication in mice [50]. It was shown that, in these AAV/1.2HBV mice, the ability of the endotoxin lipopolysaccharides (LPS) to activate the NLRP3 inflammasome and the production of IL-1β in Kupffer cells were impaired. The ability of LPS to activate the inflammasome, the cleavage of caspase-1, and the release of IL-1β from Kupffer cells were partially restored if mice were injected with the AAV/1.2HBV DNA that carried the HBeAg-null mutant [51]. It was further determined that HBeAg inhibited NF-κB and the production of reactive oxygen species (ROS) induced by LPS, thereby suppressing the activation of the NLRP3 inflammasome and the maturation and release of IL-1β. A similar reduction in IL-1β production by Kupffer cells was also observed in HBeAg-positive patients as opposed to HBeAg-negative HBV patients [51]. These findings were consistent with our observation that HBeAg could reprogram mitochondrial metabolism of macrophages to attenuate the production of IL-1β, as discussed above.

Programmed cell death induction: We recently found that HBeAg could also induce programmed cell death of macrophages [38]. This leads to a reduction in macrophage population, which likely contributes to immune escape and HBV persistence. HBeAg can induce both pyroptosis and apoptosis of M1 macrophages (Figure 1). However, it induces only apoptosis of M2 macrophages [38]. The inability of HBeAg to induce pyroptosis of M2 macrophages might be due to its suppressive effect on the activation of inflammasomes, as discussed above.

##### Dendritic Cells

Dendritic cells (DCs), which play a pivotal role in bridging innate and adaptive immunity, are subject to multifaceted regulation by HBeAg during HBV infection. By using mice as a model, HBeAg was shown to diminish the T cell stimulatory activity of DCs in a manner partially dependent on IL-10. It also reduced the ability of LPS to induce IL-12p70 in DCs, apparently by activating the PI3K-Akt pathway [52] (Figure 1). In addition, the treatment of mouse bone marrow cells with HBeAg led to a dose-dependent reduction in CD11c^+^ DCs and an increase in CD11b^+^Ly6G^+^ immature myeloid cells, suggesting that HBeAg could impair the differentiation of bone marrow progenitor cells into CD11c^+^ DCs [53]. HBeAg also reduced the ability of plasmacytoid DCs (pDCs) to produce interferon-α (IFN-α) induced by CpG, a TLR9 agonist. However, it had no effect on IFN-α production induced by Loxoribine, a TLR7 agonist, indicating a TLR9-specific suppressive effect by HBeAg. This observation was confirmed by the analysis of pDCs isolated from chronic HBV carriers. It was found that pDCs isolated from HBeAg-positive patients had significantly reduced ability to produce IFN-α than those isolated from HBeAg-negative patients or healthy controls when they were treated with CpG ex vivo, but not when they were treated with Loxoribine [54]. These effects of HBeAg on pDCs likely facilitate HBV immune evasion and persistence [54].

##### Natural Killer Cells

Natural killer (NK) cells can suppress HBV replication. It was found that the expression of the activating receptor NKp46 in NK cells was negatively correlated with HBV DNA levels in patients [54]. However, as NK cells from chronic HBV patients with high viral load displayed lower cytolytic activities, HBV could apparently also regulate the NK cell activity [55]. HBeAg did not affect the functions of NK cells when it was used to treat human peripheral blood mononuclear cells (PBMCs) ex vivo [56]. However, it could indirectly suppress NK cell activities via the activation of neutrophils. HBeAg positivity was associated with the activation of neutrophils in chronic HBV patients, and HBeAg could activate neutrophils ex vivo [56]. If HBeAg-activated neutrophils were co-cultured with PBMCs, NK cells would express an increased level of PD-1 with a concomitant decrease in the expression of IFN-γ and TNF-α. The blockade of the interaction between PD-1 and PD-L1 with the anti-PD-L1 antibody partially restored the expression levels of IFN-γ and TNF-α in NK cells. These results indicated that HBeAg-activated neutrophils suppressed the activities of NK cells partially via the PD-1/PD-L1 interaction [56] (Figure 1). In a separate study, it was shown that HBeAg could downregulate IL-18-induced expression of IFN-γ in NK cells of healthy donors or the established NK cell line NK92 [57]. In NK92 cells, HBeAg was shown to suppress the IFN-α-induced activation of NK cells by suppressing the activation of STAT1 and p38 MAPK [58]. HBeAg could also stimulate regulatory T cells (Tregs) to secrete IL-10, which upregulated the expression of the inhibitory receptor NKG2A on NK cells and induced their functional impairment [59,60]. In patients receiving the antiviral drug telbivudine, the HBeAg seroconversion was accompanied by a marked restoration of CD56^bright^ NK cell number with an increased expression of the activating receptor NKG2D and an associated increase in the serum level of IL-15. Telbivudine also enhanced the expression of NKG2D and IL-15 in NK cells isolated from untreated HBeAg-positive chronic HBV patients, indicating a close association of NK cell functionality with HBeAg [61]. HBeAg was also shown to reduce the NK cell population in mice injected with the 1.3mer overlength HBV genome that was cloned in an AAV vector [62]. This might also contribute to immune evasion and persistence of HBV in this mouse model. In summary, HBeAg systematically impairs the number, activity, and signaling pathways of NK cells through multiple targets and mechanisms, thereby significantly suppressing their innate immune functions and strongly promoting HBV immune evasion and persistent infection.

#### 2.2.4. Suppression of Adaptive Immune Cells

##### T Cells

In chronic HBV patients, circulating HBeAg is a key driver of dysfunction in HBV core-specific CD8^+^ T cells [34]. It is associated with increased expression levels of PD-1 and CTLA-4, two immune checkpoint receptors, in HBV-specific CD8^+^ T cells and reduced HBV core-specific T cell responses [34,63,64]. The analysis of PBMCs isolated from chronic HBV patients revealed that the blockade of PD-1 or CTLA-4 increased T cell response to HBV, although this response was weaker with HBeAg-positive patients than with HBeAg-negative patients [64]. Moreover, HBeAg-positive status was found to be associated with a reduced frequency of HBV-specific IFN-γ^+^ CD4^+^ T cells, and HBeAg loss led to an increase in these IFN-γ^+^ CD4^+^ T cells, suggesting that HBeAg delayed viral clearance by impairing Th1 cell differentiation [65]. Studies also showed that HBeAg-negative chronic HBV patients had an increased number of peripheral CD8^+^ T cells and an expanded population of IFN-γ-producing CD4^+^ and CD8^+^T cells [66]. These results together indicated that HBeAg likely played an important role in T cell exhaustion and the reduction in the intensity of T cell responses to HBV.

##### B Cells and MDSCs

In chronic HBV infection, HBeAg contributes to peripheral immune tolerance not only by impairing T cell responses but also by modulating humoral immunity and myeloid suppressor networks. Follicular helper T (Tfh) cells, which are characterized by a high expression level of chemokine receptor 5 (CXCR5), are a CD4^+^ T cell subset in B cell follicles and are essential for B cells to develop into antibody-producing cells [67]. In contrast, follicular regulatory T (Tfr) cells inhibit B cell responses. In HBeAg-positive chronic HBV carriers, the Tfr/Tfh ratio is higher than that in HBeAg-negative patients [68]. HBeAg positivity is also associated with a higher number of circulating IL-10^+^ regulatory B (Breg) cells [68], which can suppress HBV-specific CD8^+^ T cell responses in an IL-10-dependent manner [69]. The loss of HBeAg during chronic HBV infection is associated with a higher level of CXCR5^+^CD4^+^ T cells and CD19^+^CD38^+^ B cells [70]. Together, these findings suggest an interesting interplay between HBeAg, Tfh cells, and B cells, which might skew B cell responses to promote HBV persistence.

In an earlier longitudinal cohort study conducted on infants born to HBeAg-positive mothers and HBeAg-negative mothers, no statistical difference in the anti-HBsAg titers was observed between these two groups of infants 7–12 months after neonatal vaccination against HBsAg [71]. However, in a subsequent study with a much bigger cohort, infants born to HBeAg-positive mothers developed significantly lower anti-HBsAg titers than those born to HBeAg-negative mothers at 7–12 months after vaccination. No statistical difference in the antibody titers was observed at 24 months in this later study, indicating the resolution of transient immune suppression [72]. This immunological recovery corresponds with the natural decline of maternally derived HBeAg in infants, which typically becomes undetectable in the serum by 6 months postpartum [23,73]. This clinical study indicated that maternal-derived HBeAg might also suppress B cell immune response to HBsAg in infants.

Besides T cells and B cells, HBeAg also exerts potent immunosuppressive effects by inducing the expansion of monocytic myeloid-derived suppressor cells (mMDSCs) [74]. By studying chronic HBV carriers, it was found that HBeAg^+^ patients had significantly higher frequencies of circulating mMDSCs compared to HBeAg^−^ individuals. HBeAg was also found to induce the expansion of mMDSC via IL-6 and IL-1β in vitro, and these HBeAg-induced mMDSCs suppressed the proliferation of autologous T cells and IFN-γ production in a manner dependent on indoleamine 2,3-dioxygenase (IDO) [74].

Collectively, these observations support a model wherein HBeAg orchestrates peripheral immune containment by shaping a suppressive milieu through the expansion of mMDSCs and the inhibition of B cell differentiation. Although whether HBeAg has any direct effect on B cells remains to be determined, current evidence underscores its indirect regulatory role at the innate–adaptive immune interface, thereby impairing both cellular and humoral immune responses to promote chronic HBV persistence.

## 3. MTCT Risk Stratification in HBeAg-Positive Pregnancies

### 3.1. Global Disease Burden

Despite progress in HBV prevention, the timely administration of the hepatitis B vaccine birth dose (i.e., within 24 h of birth) remains suboptimal globally. According to recent estimates, only about 45% of newborns receive the timely birth dose, significantly impeding the progress toward the WHO’s 2030 target of 90% timely coverage [75,76]. Consequently, the global prevalence of chronic HBV infection in children under five years of age remains at 0.7%, significantly above the WHO elimination threshold of ≤0.1% [77].

### 3.2. Risk Stratification and Transmission Pathways

The MTCT risk of HBV varies significantly due to variations in maternal virological profiles, particularly those related to serum HBV DNA load and the HBeAg status. In regions with limited access to HBV DNA testing, HBeAg can serve as a reliable surrogate marker for identifying high-risk pregnancies [78,79,80]. According to the latest EASL and APASL Clinical Practice Guidelines, in regions where HBV DNA testing is unavailable, HBeAg-positive pregnant women should receive interventions to prevent mother-to-child transmission of HBV regardless of their HBV DNA levels [79,81]. This is particularly important, as a 2021 meta-analysis found that maternal HBeAg positivity predicted high viral load (≥200,000 IU/mL) with a pooled sensitivity of 88.2% and specificity of 92.6%, and predicted MTCT infection despite infant immunoprophylaxis with a pooled sensitivity of 99.5% and specificity of 62.2% [82]. The estimated MTCT risks under different clinical profiles are summarized in Table 1 [30,83,84].

## 4. Clinical Strategies to Prevent MTCT in HBeAg-Positive Pregnancies

### 4.1. Neonatal Immunoprophylaxis

**Figure 2:**
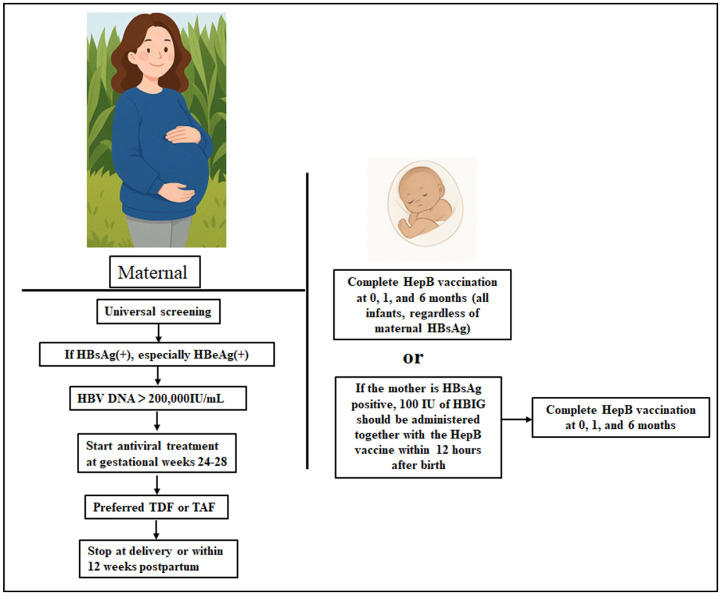
Comprehensive prevention strategy for the interruption of MTCT of HBV in HBeAg-positive pregnancies. HBsAg(+), HBsAg-positive; HBeAg(+), HBeAg-positive.

In HBeAg-positive pregnant women, early neonatal immunoprophylaxis remains the cornerstone of MTCT prevention [72,78,80,83]. The standard protocol involves administering a HBsAg vaccine prepared either in yeast (10 μg) or Chinese hamster ovary cells (20 μg) together with 100 IU of HBIG within 12 h of birth, followed by additional vaccination at 1 and 6 months for completion of the vaccination schedule [85,86]. This strategy is illustrated in Figure 2. This combined approach significantly reduces the risk of transmission from approximately 80–90% to 10–15% in high-risk infants [87,88,89]. Large-scale implementation efforts, such as China’s “SHIELD Project” (*xiao bei ke*), have reduced MTCT rates to as low as 0.23% [90].

Global guidelines from the World Health Organization (WHO) and the U.S. Centers for Disease Control and Prevention (CDC) affirm that timely hepatitis B vaccination alone can prevent approximately 75% of perinatal infections, and the addition of HBIG increases protection to about 94% [91]. Collectively, these findings support a comprehensive immunoprophylaxis strategy as the first-line defense against HBV MTCT in HBeAg-positive pregnancies. Unfortunately, there are notable regional differences in the implementation of vaccination programs. According to the 2024 WHO/UNICEF estimates, timely birth-dose coverage (≤24 h) is approximately 79% in the Western Pacific Region (WPRO) [75], 44% in the European Region (EURO) [92], and 17% in the African Region (AFRO) [75]. In the Region of the Americas (AMR), although hepatitis B vaccination is included in all national immunization schedules, only about two-thirds (34 of 51, 67%) [93] of countries have adopted a nationwide universal birth-dose policy, resulting in heterogeneous coverage within the region. These differences reflect variability in policy adoption, health-system capacity, and delivery context.

Despite the high efficacy of combined immunoprophylaxis, vaccine failure can still occur in certain circumstances. Premature and low birth weight infants often exhibit lower seroprotection rates and reduced anti-HB antibody titers following timely vaccination, likely due to immaturity of the immune system, reduced antigen-presenting cell function, and impaired antibody production [94,95,96]. Delayed administration of the birth dose beyond 24 h, improper vaccine storage or dosing errors, and extremely high maternal HBV DNA levels (>10^7^ IU/mL) have also been identified as important contributors to immunoprophylaxis failure [76,97,98]. Therefore, ensuring timely vaccination, strict quality control of vaccine storage and delivery, and close post-vaccination serologic monitoring of high-risk infants are essential to maximize the effectiveness of neonatal immunoprophylaxis.

### 4.2. Maternal Antiviral Therapy During Pregnancy

Randomized controlled trials have consistently demonstrated that maternal use of the antiviral drug tenofovir disoproxil fumarate (TDF) or tenofovir alafenamide (TAF), in conjunction with neonatal HBIG and vaccination, further enhances protective efficacy [99,100]. Antiviral therapy is recommended for all pregnant women with serum HBV DNA levels ≥ 200,000 IU/mL, irrespective of HBeAg status, in the third trimester of pregnancy. Maternal antiviral therapy during pregnancy is strongly recommended to reduce the risk of MTCT (Figure 2), particularly in cases where high-level viremia may lead to prophylaxis failure despite standard neonatal immunization [100]. Antiviral treatment is typically initiated between gestational weeks 24 and 28 and can be started as early as week 24 in women with extremely high viral load (>10^9^ IU/mL) or a history of prophylaxis failure. The use of TDF, a nucleoside analog, led to approximately 80% reduction in MTCT risk and a favorable maternal and neonatal safety profile [101,102]. More recently, TAF was also shown to achieve a near-zero MTCT rate when administered during the second or early third trimester of pregnancy [100]. Notably, a 2023 multicenter cohort study in Asia reported a 0.0% transmission rate among 139 infants born to TAF-treated mothers, with no adverse outcomes at 12 months postpartum [103]. Unless long-term treatment is clinically indicated (e.g., in women with cirrhosis or persistent high viral load), antiviral therapy can generally be discontinued at delivery or within 12 weeks postpartum. Collectively, these findings underscore the importance of maternal antiviral therapy in the prevention of MTCT of HBV for HBeAg-positive pregnancies.

## 5. Unresolved Issues and Future Directions

### 5.1. Clinical Challenges

Despite well-established guidelines, several challenges continue to hinder the complete elimination of MTCT of HBV in HBeAg-positive pregnancies. One major barrier is the low global coverage of timely vaccination after birth, which remains at approximately 45%, with rates in the WHO African Region as low as 18% [104]. Since 90% of high-risk pregnant women with HBV DNA ≥ 200,000 IU/mL undergo the maternal antiviral therapy once they are identified and appropriately counselled, the primary limitation in the control of MTCT of HBV apparently lies in the testing and identification of these patients rather than patient adherence [105].

The residual risk of MTCT in high-viremia pregnancies remains a concern. Even with optimal neonatal immunoprophylaxis, the transmission risk remains around 3% for mothers with HBV DNA levels near 10^6^ IU/mL, and can increase to 7–8% at higher viral load [106]. This underscores the need for more effective maternal or combined interventions in cases of extreme viremia. In addition, the long-term safety profile of TAF, while favorable in the short to moderate term, requires further validation. Current data indicate normal growth and neurodevelopment in TAF-exposed infants up to 12 months of age [101,107]. However, data beyond the first year are lacking. The absence of long-term neurocognitive and metabolic follow-up beyond two years remains a knowledge gap, as highlighted in the WHO 2023 HBV Guidelines [108], and warrants continuous postnatal monitoring in future studies.

### 5.2. Future Strategies and Innovations

To overcome current limitations, several innovative therapeutic strategies are being explored. One promising direction involves direct targeting of HBeAg through molecular approaches such as siRNA-based therapies. In murine models, nanoparticles guided by a peptide consisting of amino acids 2-21 of the preS sequence (PreS/2-21), which binds to the HBV receptor sodium taurocholate cotransporting polypeptide (NTCP) on hepatocytes, were used to deliver siRNA targeting HBV sequences to hepatocytes. This approach successfully reduced HBeAg levels by ~55%, along with concurrent decreases in HBsAg (~39%), cccDNA (~29%), and HBV DNA (~24%), demonstrating the potential to suppress multiple viral components simultaneously [109]. In addition to this PreS/2-21 nanoparticle approach, other approaches are also being developed to deliver siRNA to suppress HBV gene expression [110,111]. In addition to siRNA, immunomodulators combined with antivirals have also been used to break immune tolerance and restore antiviral immunity. TLR agonists, such as CpG oligodeoxynucleotides (CpG ODNs), were shown to have dual antiviral and immune-enhancing activity in preclinical studies. These agents reduced serum levels of HBsAg, HBeAg, and HBV DNA while stimulating innate immunity by inducing IFN-α and IFN-γ. They can potentially be used to prevent or break immune tolerance after the MTCT of HBV [112].

Finally, other therapeutic approaches are also being explored. One of them is the use of the gene-editing technology CRISPR/Cas9 to disrupt or silence the HBV cccDNA, which has been difficult to eliminate from HBV patients [113]. Additional novel anti-HBV agents, such as core assembly modulators, are also under investigation for their ability to suppress HBV replication. Together, these emerging strategies hold great promise for improving the current antiviral therapies for the eventual goal of HBV elimination.

## 6. Conclusions

MTCT is the most important cause of chronic HBV infection, and HBeAg, which regulates both innate and adaptive immunity, plays a central role in the induction of immune tolerance. To effectively interrupt this transmission route, a combination of maternal antiviral therapy initiated in the second or third trimester based on HBV DNA ≥ 200,000 IU/mL or HBeAg positivity, together with timely neonatal immunoprophylaxis, has been used. Multi-pronged approaches are also being explored to improve the current antiviral drugs for the treatment of chronic HBV infection. These novel approaches open promising avenues for breaking HBV tolerance and the eventual elimination of HBV.

## Figures and Tables

**Table 1:** Estimated MTCT risk based on maternal virological profile.

Maternal Virological Profile	Estimated MTCT Risk			Notes
	No Intervention	HBIG + Vaccine Only	HBIG + Vaccine with Antiviral Therapy	
HBV DNA > 2 × 10^5^ IU/mL (Regardless of HBeAg)	70–90%	~10%	<1%	Strong recommendation for antiviral (TDF) therapy starting at 28–32 weeks
HBV DNA ≤ 2 × 10^5^ IU/mL and HBeAg (+) ^a^	Moderate (~10–30%)	<10&	<1%	HBeAg can serve as a surrogate marker when HBV DNA is not available
HBV DNA ≤ 2 × 10^5^ IU/mL and HBeAg (−) ^a^	Low (<5%)	<5%	Not recommended	Routine neonatal immunoprophylaxis suffice

^a^ HBeAg (+): HBeAg-positive; HBeAg (−): HBeAg-negative.

## Data Availability

No new data were created or analyzed in this study. Data sharing is not applicable to this article.

## References

[1] The Polaris Observatory Collaborators (2023). Global prevalence, cascade of care, and prophylaxis coverage of hepatitis B in 2022: A modelling study. Lancet Gastroenterol. Hepatol..

[2] Tsai K.N., Ou J.J. (2021). Hepatitis B virus e antigen and viral persistence. Curr. Opin. Virol..

[3] Beasley R.P., Trepo C., Stevens C.E., Szmuness W. (1977). The e antigen and vertical transmission of hepatitis B surface antigen. Am. J. Epidemiol..

[4] Okada K., Kamiyama I., Inomata M., Imai M., Miyakawa Y. (1976). e antigen and anti-e in the serum of asymptomatic carrier mothers as indicators of positive and negative transmission of hepatitis B virus to their infants. N. Engl. J. Med..

[5] Su W.J., Chen S.F., Yang C.H., Chuang P.H., Chang H.F., Chang M.H. (2019). The Impact of Universal Infant Hepatitis B Immunization on Reducing the Hepatitis B Carrier Rate in Pregnant Women. J. Infect. Dis..

[6] Ou J.H., Laub O., Rutter W.J. (1986). Hepatitis B virus gene function: The precore region targets the core antigen to cellular membranes and causes the secretion of the e antigen. Proc. Natl. Acad. Sci. USA.

[7] Ito K., Kim K.H., Lok A.S., Tong S. (2009). Characterization of genotype-specific carboxyl-terminal cleavage sites of hepatitis B virus e antigen precursor and identification of furin as the candidate enzyme. J. Virol..

[8] DiMattia M.A., Watts N.R., Stahl S.J., Grimes J.M., Steven A.C., Stuart D.I., Wingfield P.T. (2013). Antigenic switching of hepatitis B virus by alternative dimerization of the capsid protein. Structure.

[9] Lamberts C., Nassal M., Velhagen I., Zentgraf H., Schroder C.H. (1993). Precore-mediated inhibition of hepatitis B virus progeny DNA synthesis. J. Virol..

[10] Guidotti L.G., Matzke B., Pasquinelli C., Shoenberger J.M., Rogler C.E., Chisari F.V. (1996). The hepatitis B virus (HBV) precore protein inhibits HBV replication in transgenic mice. J. Virol..

[11] Chuang Y.C., Ou J.H. (2025). Effects of cellular membranes and the precore protein on hepatitis B virus core particle assembly and DNA replication. mBio.

[12] Eren E., Watts N.R., Dearborn A.D., Palmer I.W., Kaufman J.D., Steven A.C., Wingfield P.T. (2018). Structures of Hepatitis B Virus Core- and e-Antigen Immune Complexes Suggest Multi-point Inhibition. Structure.

[13] Takahashi K., Machida A., Funatsu G., Nomura M., Usuda S., Aoyagi S., Tachibana K., Miyamoto H., Imai M., Nakamura T. (1983). Immunochemical structure of hepatitis B e antigen in the serum. J. Immunol..

[14] Gerlich W.H., Glebe D., Kramvis A., Magnius L.O. (2020). Peculiarities in the designations of hepatitis B virus genes, their products, and their antigenic specificities: A potential source of misunderstandings. Virus Genes.

[15] Chang C., Enders G., Sprengel R., Peters N., Varmus H.E., Ganem D. (1987). Expression of the precore region of an avian hepatitis B virus is not required for viral replication. J. Virol..

[16] Realdi G., Alberti A., Rugge M., Bortolotti F., Rigoli A.M., Tremolada F., Ruol A. (1980). Seroconversion from hepatitis B e antigen to anti-HBe in chronic hepatitis B virus infection. Gastroenterology.

[17] Milich D., Liang T.J. (2003). Exploring the biological basis of hepatitis B e antigen in hepatitis B virus infection. Hepatology.

[18] Huang T.H., Zhang Q.J., Xie Q.D., Zeng L.P., Zeng X.F. (2005). Presence and integration of HBV DNA in mouse oocytes. World J. Gastroenterol..

[19] Hu X.L., Zhou X.P., Qian Y.L., Wu G.Y., Ye Y.H., Zhu Y.M. (2011). The presence and expression of the hepatitis B virus in human oocytes and embryos. Hum. Reprod..

[20] Zhong Y., Liu D.L., Ahmed M.M.M., Li P.H., Zhou X.L., Xie Q.D., Xu X.Q., Han T.T., Hou Z.W., Huang J.H. (2018). Transcription and regulation of hepatitis B virus genes in host sperm cells. Asian J. Androl..

[21] Ali B.A., Huang T.H., Salem H.H., Xie Q.D. (2006). Expression of hepatitis B virus genes in early embryonic cells originated from hamster ova and human spermatozoa transfected with the complete viral genome. Asian J. Androl..

[22] Nie R., Jin L., Zhang H., Xu B., Chen W., Zhu G. (2011). Presence of hepatitis B virus in oocytes and embryos: A risk of hepatitis B virus transmission during in vitro fertilization. Fertil. Steril..

[23] Wang Z., Zhang J., Yang H., Li X., Wen S., Guo Y., Sun J., Hou J. (2003). Quantitative analysis of HBV DNA level and HBeAg titer in hepatitis B surface antigen positive mothers and their babies: HBeAg passage through the placenta and the rate of decay in babies. J. Med. Virol..

[24] Wang J.S., Zhu Q.R. (2000). Infection of the fetus with hepatitis B e antigen via the placenta. Lancet.

[25] Huang M., Gao Y., Liao D., Li J., Tang B., Ma Y., Yin X., Li Y., Liu Z. (2020). Effects of intrauterine exposure to maternal-derived HBeAg on T cell immunity in cord blood. Scand. J. Immunol..

[26] Inaba N., Ijichi M., Ohkawa R., Takamizawa H. (1984). Placental transmission of hepatitis B e antigen and clinical significance of hepatitis B e antigen titers in children born to hepatitis B e antigen-positive carrier women. Am. J. Obstet. Gynecol..

[27] Arakawa K., Tsuda F., Takahashi K., Ise I., Naito S., Kosugi E., Miyakawa Y., Mayumi M. (1982). Maternofetal transmission of IgG-bound hepatitis B e antigen. Pediatr. Res..

[28] Lin H.H., Lee T.Y., Chen D.S., Sung J.L., Ohto H., Etoh T., Kawana T., Mizuno M. (1987). Transplacental leakage of HBeAg-positive maternal blood as the most likely route in causing intrauterine infection with hepatitis B virus. J. Pediatr..

[29] Du X., Zhang L., Liu Z., Qian Y., Zhang X., Hu T., Liu S., Wang H., Zhang Z. (2024). Risk of mother-to-child transmission after amniocentesis in pregnant women with hepatitis B virus: A retrospective cohort study. Am. J. Obstet. Gynecol..

[30] Pan C.Q., Duan Z.P., Bhamidimarri K.R., Zou H.B., Liang X.F., Li J., Tong M.J. (2012). An algorithm for risk assessment and intervention of mother to child transmission of hepatitis B virus. Clin. Gastroenterol. Hepatol..

[31] Pan Y.C., Jia Z.F., Wang Y.Q., Yang N., Liu J.X., Zhai X.J., Song Y., Wang C., Li J., Jiang J. (2020). The role of caesarean section and nonbreastfeeding in preventing mother-to-child transmission of hepatitis B virus in HBsAg-and HBeAg-positive mothers: Results from a prospective cohort study and a meta-analysis. J. Viral Hepat..

[32] Lin H.H., Hsu H.Y., Chang M.H., Chen P.J., Chen D.S. (1993). Hepatitis B virus in the colostra of HBeAg-positive carrier mothers. J. Pediatr. Gastroenterol. Nutr..

[33] Hong M., Sandalova E., Low D., Gehring A.J., Fieni S., Amadei B., Urbani S., Chong Y.S., Guccione E., Bertoletti A. (2015). Trained immunity in newborn infants of HBV-infected mothers. Nat. Commun..

[34] Chen M.T., Billaud J.N., Sallberg M., Guidotti L.G., Chisari F.V., Jones J., Hughes J., Milich D.R. (2004). A function of the hepatitis B virus precore protein is to regulate the immune response to the core antigen. Proc. Natl. Acad. Sci. USA.

[35] Lee B.O., Tucker A., Frelin L., Sallberg M., Jones J., Peters C., Hughes J., Whitacre D., Darsow B., Peterson D.L. (2009). Interaction of the hepatitis B core antigen and the innate immune system. J. Immunol..

[36] Murray P.J., Wynn T.A. (2011). Protective and pathogenic functions of macrophage subsets. Nat. Rev. Immunol..

[37] Martinez F.O., Gordon S. (2014). The M1 and M2 paradigm of macrophage activation: Time for reassessment. F1000Prime Rep..

[38] Li Y., Wu C., Lee J., Ning Q., Lim J., Eoh H., Wang S., Hurrell B.P., Akbari O., Ou J.J. (2024). Hepatitis B virus e antigen induces atypical metabolism and differentially regulates programmed cell deaths of macrophages. PLoS Pathog..

[39] Tian Y., Kuo C.F., Akbari O., Ou J.H. (2016). Maternal-Derived Hepatitis B Virus e Antigen Alters Macrophage Function in Offspring to Drive Viral Persistence after Vertical Transmission. Immunity.

[40] Mills E.L., Kelly B., Logan A., Costa A.S.H., Varma M., Bryant C.E., Tourlomousis P., Däbritz J.H.M., Gottlieb E., Latorre I. (2016). Succinate Dehydrogenase Supports Metabolic Repurposing of Mitochondria to Drive Inflammatory Macrophages. Cell.

[41] Tannahill G.M., Curtis A.M., Adamik J., Palsson-McDermott E.M., McGettrick A.F., Goel G., Frezza C., Bernard N.J., Kelly B., Foley N.H. (2013). Succinate is an inflammatory signal that induces IL-1β through HIF-1α. Nature.

[42] Liu Y., Xu R., Gu H., Zhang E., Qu J., Cao W., Huang X., Yan H., He J., Cai Z. (2021). Metabolic reprogramming in macrophage responses. Biomark. Res..

[43] Li Y., Zhu Y., Feng S., Ishida Y., Chiu T.P., Saito T., Wang S., Ann D.K., Ou J.J. (2022). Macrophages activated by hepatitis B virus have distinct metabolic profiles and suppress the virus via IL-1β to downregulate PPARα and FOXO3. Cell Rep..

[44] Watashi K., Liang G., Iwamoto M., Marusawa H., Uchida N., Daito T., Kitamura K., Muramatsu M., Ohashi H., Kiyohara T. (2013). Interleukin-1 and tumor necrosis factor-alpha trigger restriction of hepatitis B virus infection via a cytidine deaminase activation-induced cytidine deaminase (AID). J. Biol. Chem..

[45] Wang W., Bian H., Li F., Li X., Zhang D., Sun S., Song S., Zhu Q., Ren W., Qin C. (2018). HBeAg induces the expression of macrophage miR-155 to accelerate liver injury via promoting production of inflammatory cytokines. Cell Mol. Life Sci..

[46] Tian M., Wu N., Xie X., Liu T., You Y., Ma S., Bian H., Cao H., Wang L., Liu C. (2024). Phosphorylation of RGS16 at Tyr168 promote HBeAg-mediated macrophage activation by ERK pathway to accelerate liver injury. J. Mol. Med..

[47] Sun L., Yu J., Zhang N., Wang Y., Qi J. (2023). M1 macrophages may be effective adjuvants for promoting Th-17 differentiation in HBeAg positive hepatitis patients with ALT ≤ 2ULN. Mol. Med. Rep..

[48] Li J., Yu M., Zong R., Fan C., Ren F., Wu W., Li C. (2022). Deacetylation of Notch1 by SIRT1 contributes to HBsAg- and HBeAg-mediated M2 macrophage polarization. Am. J. Physiol. Gastrointest. Liver Physiol..

[49] Bility M.T., Cheng L., Zhang Z., Luan Y., Li F., Chi L., Zhang L., Tu Z., Gao Y., Fu Y. (2014). Hepatitis B virus infection and immunopathogenesis in a humanized mouse model: Induction of human-specific liver fibrosis and M2-like macrophages. PLoS Pathog..

[50] Huang L.R., Wu H.L., Chen P.J., Chen D.S. (2006). An immunocompetent mouse model for the tolerance of human chronic hepatitis B virus infection. Proc. Natl. Acad. Sci. USA.

[51] Yu X., Lan P., Hou X., Han Q., Lu N., Li T., Jiao C., Zhang J., Zhang C., Tian Z. (2017). HBV inhibits LPS-induced NLRP3 inflammasome activation and IL-1β production via suppressing the NF-κB pathway and ROS production. J. Hepatol..

[52] Lan S., Wu L., Wang X., Wu J., Lin X., Wu W., Huang Z. (2016). Impact of HBeAg on the maturation and function of dendritic cells. Int. J. Infect. Dis..

[53] Hatipoglu I., Ercan D., Acilan C., Basalp A., Durali D., Baykal A.T. (2014). Hepatitis B virus e antigen (HBeAg) may have a negative effect on dendritic cell generation. Immunobiology.

[54] Woltman A.M., Op den Brouw M.L., Biesta P.J., Shi C.C., Janssen H.L. (2011). Hepatitis B virus lacks immune activating capacity, but actively inhibits plasmacytoid dendritic cell function. PLoS ONE.

[55] Li W., Jiang Y., Wang X., Jin J., Qi Y., Chi X., Zhang H., Feng X., Niu J. (2015). Natural Killer p46 Controls Hepatitis B Virus Replication and Modulates Liver Inflammation. PLoS ONE.

[56] Feng Z., Fu J., Tang L., Bao C., Liu H., Liu K., Yang T., Yuan J.H., Zhou C.B., Zhang C. (2024). HBeAg induces neutrophils activation impairing NK cells function in patients with chronic hepatitis B. Hepatol. Int..

[57] Jegaskanda S., Ahn S.H., Skinner N., Thompson A.J., Ngyuen T., Holmes J., De Rose R., Navis M., Winnall W.R., Kramski M. (2014). Downregulation of interleukin-18-mediated cell signaling and interferon gamma expression by the hepatitis B virus e antigen. J. Virol..

[58] Yang Y., Han Q., Zhang C., Xiao M., Zhang J. (2016). Hepatitis B virus antigens impair NK cell function. Int. Immunopharmacol..

[59] Ma Q., Dong X., Liu S., Zhong T., Sun D., Zong L., Zhao C., Lu Q., Zhang M., Gao Y. (2020). Hepatitis B e Antigen Induces NKG2A(+) Natural Killer Cell Dysfunction via Regulatory T Cell-Derived Interleukin 10 in Chronic Hepatitis B Virus Infection. Front. Cell Dev. Biol..

[60] Tang R., Lei Z., Wang X., Qi Q., He J., Liu D., Wang X., Chen X., Zhu J., Li Y. (2020). Hepatitis B envelope antigen increases Tregs by converting CD4+CD25(-) T cells into CD4(+)CD25(+)Foxp3(+) Tregs. Exp. Ther. Med..

[61] Chen T., Zhu L., Shi A., Ding L., Zhang X., Tan Z., Guo W., Yan W., Han M., Jia J. (2017). Functional restoration of CD56(bright) NK cells facilitates immune control via IL-15 and NKG2D in patients under antiviral treatment for chronic hepatitis B. Hepatol. Int..

[62] Zhang J.M., Kang N.L., Wu L.Y., Zeng D.W. (2023). Hepatitis B Virus Envelope Antigen and Hepatitis B Virus Surface Antigen Both Contribute to the Innate Immune Response During Persistent Hepatitis B Virus Infection. Viral Immunol..

[63] Peng G., Luo B., Li J., Zhao D., Wu W., Chen F., Chen Z. (2011). Hepatitis B e-antigen persistency is associated with the properties of HBV-specific CD8 T cells in CHB patients. J. Clin. Immunol..

[64] Park J.J., Wong D.K., Wahed A.S., Lee W.M., Feld J.J., Terrault N., Khalili M., Sterling R.K., Kowdley K.V., Bzowej N. (2016). Hepatitis B Virus--Specific and Global T-Cell Dysfunction in Chronic Hepatitis B. Gastroenterology.

[65] Wang H., Luo H., Wan X., Fu X., Mao Q., Xiang X., Zhou Y., He W., Zhang J., Guo Y. (2020). TNF-α/IFN-γ profile of HBV-specific CD4 T cells is associated with liver damage and viral clearance in chronic HBV infection. J. Hepatol..

[66] Vassilopoulos D., Rapti I., Nikolaou M., Hadziyannis E., Hadziyannis S.J. (2008). Cellular immune responses in hepatitis B virus e antigen negative chronic hepatitis B. J. Viral Hepat..

[67] Crotty S. (2011). Follicular helper CD4 T cells (TFH). Annu. Rev. Immunol..

[68] Wang L., Qiu J., Yu L., Hu X., Zhao P., Jiang Y. (2014). Increased numbers of CD5^+^CD19^+^CD1dhighIL-10^+^ Bregs, CD4^+^Foxp3^+^ Tregs, CD4^+^CXCR5^+^Foxp3^+^ follicular regulatory T (TFR) cells in CHB or CHC patients. J. Transl. Med..

[69] Das A., Ellis G., Pallant C., Lopes A.R., Khanna P., Peppa D., Chen A., Blair P., Dusheiko G., Gill U. (2012). IL-10-producing regulatory B cells in the pathogenesis of chronic hepatitis B virus infection. J. Immunol..

[70] Zhang L., Zhang M., Li H., Chen Z., Luo A., Liu B., Chen M., Peng M., Ren H., Hu P. (2016). Tfh cell-mediated humoral immune response and HBsAg level can predict HBeAg seroconversion in chronic hepatitis B patients receiving peginterferon-α therapy. Mol. Immunol..

[71] Huang H., Ning M., Liu J., Chen J., Feng J., Dai Y., Hu Y., Zhou Y.H. (2019). Comparison of antibody response to hepatitis B vaccination in infants with positive or negative maternal hepatitis B e antigen (HBeAg) in cord blood: Implication for the role of HBeAg as an immunotolerogen. Hum. Vaccin. Immunother..

[72] Jiang H., Chen C., Yuan D., Ye X., Chen Y., Han G., Zhou G., Ju Y., Cao M. (2023). The relationship of maternal hepatitis B e antigen and response to vaccination of infants born to women with chronic infection. BMC Pregnancy Childbirth.

[73] Milich D.R. (2019). Is the function of the HBeAg really unknown?. Hum. Vaccin. Immunother..

[74] Yang F., Yu X., Zhou C., Mao R., Zhu M., Zhu H., Ma Z., Mitra B., Zhao G., Huang Y. (2019). Hepatitis B e antigen induces the expansion of monocytic myeloid-derived suppressor cells to dampen T-cell function in chronic hepatitis B virus infection. PLoS Pathog..

[75] UNICEF Immunization Coverage Estimates Data Visualization. https://data.unicef.org/resources/immunization-coverage-estimates-data-visualization/.

[76] Solomon-Rakiep T., Olivier J., Amponsah-Dacosta E. (2023). Weak Adoption and Performance of Hepatitis B Birth-Dose Vaccination Programs in Africa: Time to Consider Systems Complexity?-A Scoping Review. Trop. Med. Infect. Dis..

[77] Childs L., Adrien P., Minta A.A., François J., Phaïmyr Jn Charles N., Blot V., Rey-Benito G., Vanden Eng J.L., Tohme R.A. (2019). Prevalence of Chronic Hepatitis B Virus Infection among Children in Haiti, 2017. Am. J. Trop. Med. Hyg..

[78] Lu Y., Song Y., Zhai X., Zhu F., Liu J., Chang Z., Li Y., Xiao Y., Li L., Liu M. (2021). Maternal hepatitis B e antigen can be an indicator for antiviral prophylaxis of perinatal transmission of hepatitis B virus. Emerg. Microbes Infect..

[79] European Association for the Study of the Liver (2025). EASL Clinical Practice Guidelines on the management of hepatitis B virus infection. J. Hepatol..

[80] Segeral O., Dim B., Durier C., Prak S., Chhim K., Vong C., Pech S., Tiv S., Nem B., Hout K. (2020). Hepatitis B e Antigen (HBeAg) Rapid Test and Alanine Aminotransferase Level-Based Algorithm to Identify Pregnant Women at Risk of HBV Mother-to-Child Transmission: The ANRS 12345 TA PROHM Study. Clin. Infect. Dis..

[81] Kumar M., Abbas Z., Azami M., Belopolskaya M., Dokmeci A.K., Ghazinyan H., Jia J., Jindal A., Lee H.C., Lei W. (2022). Asian Pacific association for the study of liver (APASL) guidelines: Hepatitis B virus in pregnancy. Hepatol. Int..

[82] Boucheron P., Lu Y., Yoshida K., Zhao T., Funk A.L., Lunel-Fabiani F., Guingané A., Tuaillon E., van Holten J., Chou R. (2021). Accuracy of HBeAg to identify pregnant women at risk of transmitting hepatitis B virus to their neonates: A systematic review and meta-analysis. Lancet Infect. Dis..

[83] Yao N., Fu S., Wu Y., Tian Z., Feng Y., Li J., Luo X., Yang Y., Ji F., Chen Y. (2022). Incidence of mother-to-child transmission of hepatitis B in relation to maternal peripartum antiviral prophylaxis: A systematic review and meta-analysis. Acta Obstet. Gynecol. Scand..

[84] Shan S., Jia J. (2021). Prevention of Mother-to-Child Transmission of Hepatitis B Virus in the Western Pacific Region. Clin. Liver Dis..

[85] Marion S.A., Tomm Pastore M., Pi D.W., Mathias R.G. (1994). Long-term follow-up of hepatitis B vaccine in infants of carrier mothers. Am. J. Epidemiol..

[86] Liao X., Liang Z. (2015). Strategy vaccination against Hepatitis B in China. Hum. Vaccin. Immunother..

[87] Funk A.L., Lu Y., Yoshida K., Zhao T., Boucheron P., van Holten J., Chou R., Bulterys M., Shimakawa Y. (2021). Efficacy and safety of antiviral prophylaxis during pregnancy to prevent mother-to-child transmission of hepatitis B virus: A systematic review and meta-analysis. Lancet Infect. Dis..

[88] Yi P., Chen R., Huang Y., Zhou R.R., Fan X.G. (2016). Management of mother-to-child transmission of hepatitis B virus: Propositions and challenges. J. Clin. Virol..

[89] Cheung K.W., Seto M.T.Y., Lao T.T. (2019). Prevention of perinatal hepatitis B virus transmission. Arch. Gynecol. Obstet..

[90] Yin X., Wang W., Chen H., Mao Q., Han G., Yao L., Gao Q., Gao Y., Jin J., Sun T. (2024). Real-world implementation of a multilevel interventions program to prevent mother-to-child transmission of HBV in China. Nat. Med..

[91] Mast E.E., Weinbaum C.M., Fiore A.E., Alter M.J., Bell B.P., Finelli L., Rodewald L.E., Douglas J.M., Janssen R.S., Ward J.W. (2006). A comprehensive immunization strategy to eliminate transmission of hepatitis B virus infection in the United States: Recommendations of the Advisory Committee on Immunization Practices (ACIP) Part II: Immunization of adults. MMWR Recomm. Rep..

[92] WHO (2024). Immunization Dashboard: European Region.

[93] Alleman M.M., Sereno L.S., Whittembury A., Li X., Contreras M., Pacis-Tirso C., Gonzalez M.V., Broome K., Jones S., Salas D. (2024). Progress toward elimination of mother-to-child transmission of hepatitis B virus—Region of the Americas, 2012–2022. Morb. Mortal. Wkly. Rep. (MMWR).

[94] Gaudelus J., Pinquier D., Romain O., Thiebault G., Vie le Sage F., Dommergues M.A., Hau I., Bakhache P., Virey B., Dufour V. (2014). Is the new vaccination schedule recommended in France adapted to premature babies?. Arch. Pediatr..

[95] Plotkin S.A. (2001). Immunologic correlates of protection induced by vaccination. Pediatr. Infect. Dis. J..

[96] Gagneur A., Pinquier D., Quach C. (2015). Immunization of preterm infants. Hum. Vaccin. Immunother..

[97] Qin W., Wang Y., Zhang X., Pan F., Cheng K., Sui H., Xie S. (2022). A retrospective study of hepatitis B vaccination in preterm birth and low birth weight infants born to hepatitis B surface antigen-positive mothers: Time to close the policy-practice gap. Hum. Vaccin. Immunother..

[98] World Health Organization (2017). Hepatitis B vaccines: WHO position paper—July 2017. Wkly. Epidemiol. Rec..

[99] Zhou Y.-H. (2019). Issues Meriting Further Study in Preventing Mother-to-Infant Transmission of Hepatitis B by Antiviral Therapy During Pregnancy. Matern.-Fetal Med..

[100] Pan C.Q., Zhu L., Yu A.S., Zhao Y., Zhu B., Dai E. (2024). Tenofovir Alafenamide Versus Tenofovir Disoproxil Fumarate for Preventing Vertical Transmission in Chronic Hepatitis B Mothers: A Systematic Review and Meta-Analysis. Clin. Infect. Dis..

[101] Chen R., Zou J., Long L., Huang H., Zhang M., Fan X., Huang Y. (2021). Safety and Efficacy of Tenofovir Alafenamide Fumarate in Early-Middle Pregnancy for Mothers with Chronic Hepatitis B. Front. Med..

[102] Chen J.Z., Liao Z.W., Huang F.L., Su R.K., Wang W.B., Cheng X.Y., Chen J.Q., Liu J.Q., Huang Z. (2017). Efficacy and safety of tenofovir disoproxil fumarate in preventing vertical transmission of hepatitis B in pregnancies with high viral load. Sci. Rep..

[103] Zeng Q.L., Zhou Y.H., Dong X.P., Zhang J.Y., Li G.M., Xu J.H., Chen Z.M., Song N., Zhang H.X., Chen R.Y. (2025). Expected 8-Week Prenatal vs. 12-Week Perinatal Tenofovir Alafenamide Prophylaxis to Prevent Mother-to-Child Transmission of Hepatitis B Virus: A Multicenter, Prospective, Open-Label, Randomized Controlled Trial. Am. J. Gastroenterol..

[104] WHO (2024). Guidelines Approved by the Guidelines Review Committee. Guidelines for the Prevention, Diagnosis, Care and Treatment for People with Chronic Hepatitis B Infection.

[105] Hui P.W., Ng C., Cheung K.W., Lai C.L. (2020). Acceptance of antiviral treatment and enhanced service model for pregnant patients carrying hepatitis B. Hong Kong Med. J..

[106] Gentile I., Borgia G. (2014). Vertical transmission of hepatitis B virus: Challenges and solutions. Int. J. Womens Health.

[107] Wedemeyer H., Schöneweis K., Bogomolov P., Blank A., Voronkova N., Stepanova T., Sagalova O., Chulanov V., Osipenko M., Morozov V. (2023). Safety and efficacy of bulevirtide in combination with tenofovir disoproxil fumarate in patients with hepatitis B virus and hepatitis D virus coinfection (MYR202): A multicentre, randomised, parallel-group, open-label, phase 2 trial. Lancet Infect. Dis..

[108] WHO (2024). Hepatitis B. https://www.who.int/news-room/fact-sheets/detail/hepatitis-b.

[109] Gao L., Yang J., Feng J., Liu Z., Dong Y., Luo J., Yu L., Wang J., Fan H., Ma W. (2022). PreS/2-21-Guided siRNA Nanoparticles Target to Inhibit Hepatitis B Virus Infection and Replication. Front. Immunol..

[110] Van Gulck E., Conceição-Neto N., Aerts L., Pierson W., Verschueren L., Vleeschouwer M., Krishna V., Nájera I., Pauwels F. (2024). Retreatment with HBV siRNA Results in Additional Reduction in HBV Antigenemia and Immune Stimulation in the AAV-HBV Mouse Model. Viruses.

[111] Nguyen L., Nguyen T.T., Kim J.Y., Jeong J.H. (2024). Advanced siRNA delivery in combating hepatitis B virus: Mechanistic insights and recent updates. J. Nanobiotechnol..

[112] Hu W., Huang H., Zhang T.Y., Mao Y.Y., Wang X.J., Wang S.Q. (2015). CpG oligodeoxynucleotide inhibits HBV replication in a hydrodynamic injection murine model. Antivir. Ther..

[113] Cai B., Chang S., Tian Y., Zhen S. (2023). CRISPR/Cas9 for hepatitis B virus infection treatment. Immun. Inflamm. Dis..

